# TOAST Subtypes of Ischemic Stroke and Its Risk Factors: A Hospital-Based Study at Cipto Mangunkusumo Hospital, Indonesia

**DOI:** 10.1155/2018/9589831

**Published:** 2018-11-11

**Authors:** Salim Harris, Saleha Sungkar, Al Rasyid, Mohammad Kurniawan, Taufik Mesiano, Rakhmad Hidayat

**Affiliations:** ^1^Division of Neurovascular-Neurosonology-Neurointervention, Department of Neurology, Faculty of Medicine Universitas Indonesia, Jakarta, Indonesia; ^2^Department of Parasitology, Faculty of Medicine Universitas Indonesia, Jakarta, Indonesia

## Abstract

**Background and Purpose:**

Stroke is a leading cause of death and disability, with ischemic stroke as the highest prevalent cases in Indonesia. Ischemic stroke can be classified further into five subtypes according to TOAST classification. Numerous studies have revealed that stroke risk factor has variable correlation with different stroke subtype. Currently, there is no data regarding this phenomenon in Indonesia. The aim of study is to identify characteristic of ischemic stroke subtypes and the risk factors in TOAST classification.

**Methods:**

A retrospective, cross-sectional study of patients diagnosed with ischemic stroke at Cipto Mangunkusumo Hospital from January till December 2016. Demographic data, ischemic stroke subtypes, risk factors, and other relevant data were documented. Bivariate and multivariate analysis was done using SPSS 23.

**Results:**

235 recorded data patients were included. Large artery atherosclerosis (LAA) was the most prevalent stroke subtypes at 59,6%, followed with small vessel disease (SVD) at 26,7%, undetermined etiology at 9,8%, cardioembolism (CE) at 2,1%, and other determined etiology at 0,9%. Hypertension was the most common vascular risk factor. However, it was only significant in SVD (p=0,023) and undetermined etiology subtypes (p<0,001). Significant risk factor in LAA was diabetes (55%; p=0,016) while in CE subtypes was atrial fibrillation (60%;p<0,001). In multivariate analyses, hypertension (OR 3; 95% CI 1,12-8,05) was the only variable that was related to SVD while in CE it was atrial fibrillation (OR 113,5; 95% CI 13,6-946,5).

**Conclusion:**

LAA was the most common stroke ischemic subtypes. Associated risk factor in LAA was diabetes while in SVD and undetermined etiology subtypes it was hypertension. Atrial fibrillation was associated with cardioembolism.

## 1. Introduction

Stroke is the most common cause of disability and the number two cause of death worldwide [1, 2]. The number of stroke varies in different countries. It is reported that stroke increased by approximately10% in developing countries over ten consecutive years (from 1990 to 2010) [3]. Stroke is considered as one of major burden diseases in Indonesia. According to World Health Organization (WHO) data (2012), stroke was number one killer in Indonesia with percentage 21% [4].

Stroke was considered as a preventable disease with several modifiable risk factors such as hypertension, diabetes mellitus, dyslipidemia, heart disease, atrial fibrillation, smoking, body mass index (BMI), and alcoholism. A multicenter prospective cross-sectional study in Indonesia (2018) reported that most common stroke risk factors are hypertension, diabetes mellitus, and dyslipidemia. Among 5411 stroke patients, 67,03% cases were diagnosed with ischemic stroke [5].

In 1993, The Trial of Org 10172 in Acute Stroke Treatment (TOST) classification was introduced to classify ischemic stroke according to the presumed etiological mechanism. Ischemic stroke is divided into five groups: large artery atherosclerosis (LAA), small vessel disease (SVD), cardio embolic disease (CE), other determined etiology, and undetermined etiology. The TOAST classification has been proven to be valid and reliable even in retrospective study [6, 7].

Several studies had been done to investigate the risk factors in each ischemic stroke subtypes in different population. However, the results were varied among the studies [8]. Finding associated risk factors in each etiology may influence prevention, management, and prognosis of the disease [6]. There is no available data of TOAST classification and the risk factors in Indonesia. The aim of the study was to classify the characteristic of ischemic stroke and identify the risk factors among Indonesian population.

## 2. Methods

This was a retrospective, cross-sectional study conducted in Cipto Mangunkusumo Hospital (RSCM), Jakarta, which is a tertiary care and top referral hospital. Data was retrieved from electronic medical records of patients who were admitted to neurology department from January until December 2016. Out of 1056 patient, 542 patients were diagnosed with ischemic stroke. Patients who were fulfilling the diagnostic criteria for acute stroke by World Health Organization were included in the study. It is defined as clinical signs of focal or global cerebral dysfunction that lasting more than 24 hours, with no other than vascular cause [9]. Patients with old ischemic stroke, transient ischemic attacks, hemorrhagic stroke, secondary focal neurological deficit, and incomplete electronic medical record were excluded. A total of 235 patient medical records were included. Data demographics, risk factors (hypertension, dyslipidemia, diabetes mellitus, myocardial infarction history, and atrial fibrillation), and result of related investigations (clinical, laboratory and radiology examination) were documented. In this study, chest radiography, electrocardiography, brain computed tomography (CT), and/or magnetic resonance imaging were performed in all patients, while majority of the patients also underwent echocardiography, magnetic resonance angiography and/or CT angiography, carotid ultrasound Doppler (CD), and transcranial Doppler (TCD).

Ischemic strokes were classified according to the TOAST criteria into 5 categories [6, 10]: (1) large artery atherosclerosis, which is diagnosed from clinical and radiological findings of >50% occlusion or stenosis in major brain artery or branch cortical artery; (2) small vessel disease, defined as clinical lacunar syndrome (with no cortical dysfunction), while CT or MRI lesion should be less than 1,5cm; (3) cardioembolism, if there was, at least, one major cardiac risk factor for embolism with no evidence of other stroke subtypes; (4) other determined etiology, if there was evidence of other stroke risk factor (hypercoagulable states or nonatherosclerotic vasculopathy) and absence of other stroke subtypes features; (5) stroke of undetermined etiology, if there were more than one potential cause and no etiology found from the investigations. Identification of stroke subtypes was done after all recorded data had been reviewed.

Statistical analysis was performed using statistical software SPSS 23.0 for Windows. Numerical data of age is presented in mean ± standard deviation (SD) while other risk factors and subtypes are presented in frequencies and percentages. Chi-square test was performed to compare between risk factors with each TOAST subtypes. Fisher's exact test was also performed if the requirement for Chi-square test was not fulfilled. The analysis result was defined statistically significant if* p*-value <0,05. Multivariate logistic regression analysis was also performed between variables that were considered associated with stroke subtypes and variables with a* p-*value <0,2 in bivariate analysis.

## 3. Result

Of all the 235 patients, 139 (59,1%) were male and 96 (40,9%) patients were female. The mean (±SD) age was 60,9 (11,4) years, ranging between 26 and 93 years old. A total of 153 (65,1%) patients were less than equal to 65 years old. The most common stroke subtypes were large artery atherosclerosis (n=140; 59,6%), followed by small vessel disease (n=65,2;27,7%), undetermined etiology (n=23; 9,8%), cardioembolism (n=5;2,1%), and other determined etiology (n=2;0,9%). Hypertension was the most prevalent risk factor at 83,4% (n=196). Other risk factors were dyslipidemia at 50,6% (n=119), diabetes at 48,5% (n=114), myocardial infarction at 5,5% (n=13), and atrial fibrillation at 2,6% (n=6). Characteristic of the subject are described in Table 1.

Prevalence of the risk factors varied among different etiologic subtypes. In LAA subtype, the most common risk factor was hypertension in 85,3% (n=118) of patients but it was not statistically significant (p=0,66), while diabetes mellitus as the second major risk factor in 77 (55%) patients was statistically significant (p=0,016). Hypertension was also the major risk factor in small vessel disease (n=60;92,3%) and undetermined etiology (n=13;56,5%) with p=0,023 and p<0,001, respectively. In multivariate analysis of SVD subtype, hypertension was the only risk factor associated with SVD with OR 3 (95% CI 1,12-8,05). Atrial fibrillation was present in 60% (n=3) patients with cardioembolism with p<0,001. The least frequent stroke subtype was other determined etiology and there were no risk factors associated significantly in this group. Proportion of gender and ages varied among stroke subtype. The major risk factor and its significant association in each subtype are summarized in Table 2. Multivariate analysis results were described in Table 3.

## 4. Discussion

Among 1056 patients, 664 (62,9%) were diagnosed with stroke. Among the stroke patients, 81,6% of them were diagnosed with ischemic stroke (n=542), which was similar to previous study conducted by Toyoda et al. [11] in Japan and Flaherty et al. [12] in Korea. A total of 235 ischemic stroke patients were included in this study with proportion of male was greater than female patients. This may be caused by higher ischemic stroke risk factors in male such as hypertension, smoking, coronary artery disease, dyslipidemia and atrial fibrillation [13, 14]. However, the correlation between gender and ischemic stroke subtypes remains unclear. Grau et al. [15] and Forster et al. [16] concluded that gender had significant association with certain ischemic stroke subtypes, while Yu C et al. [17], Turin et al. [18], and Al Hashel et al. [19] concluded insignificant association. In this study, we also found that there was no significant association between gender and ischemic stroke subtypes.

Prevalence of ischemic stroke subtypes was varied among published studies. Result of this study showed large artery atherosclerosis in majority of patients (59,6%). Prevalence of small vessel disease, cardioembolism, other determined etiology and undetermined etiology were found in 27,7%; 2,1%; 0,9%; and 9,8% patients, respectively. The result of this study was different with previous multicenter study by Harris et al. [5] in Indonesia which reported that among 3627 patients whom had ischemic stroke, 45,07% of them (n=1635) had lacunar infarction while in our study it was only found in 27,7% of patients (n=65). The difference of the prevalence of small vessel disease was possible considering our hospital is a tertiary and top referral hospital. As the result, only referral cases were admitted to our hospital, which may not represent the whole population in Indonesia. In this study there was 0,9% stroke of other determined etiology. This number is lower than a study conducted by Arboix et al. which found 6% of brain infarct of unusual causes [20]. We identified two causes of stroke of other determined etiology, which were hypercoagulation state and infection. This was similar to Arboix et al. which found that the common unusual causes of stroke were hematologic disorders, infections, migraine stroke, cerebral venous thrombosis, primary inflammatory vascular disorders and miscellaneous disorders [20].

LAA was the most common ischemic stroke subtype in our study. It was similar with previous studies of ischemic stroke subtypes by Giroud et al. [21] in French, Marrone et al. [22] in Brazil, and Deleu et al. [23] in Arabian Gulf Countries (AGC). In other Asian countries, small vessel disease was the most prevalent ischemic stroke subtype. A review study in Taiwan reported that majority of ischemic stroke subtype was small vessel disease (37,7%) followed by large artery atherosclerosis (27,7%) [24]. Small vessel disease was also the most prevalent stroke subtype in Jordan (36%) [25], Japan (54,1%) [18], and Kuwait (69,8%) [19]. Moreover, latest retrospective study in Kingdom of Saudi Arabia by Zafar et al. showed that small vessel disease was the first etiologic ischemic stroke subtype (32,1%), followed by cardioembolism (21,9%) and large artery atherosclerosis (14,6%) [26]. However, a large multicenter study in Germany by Grau et al. [15] and a cohort study by Acciarresi et al. [27] had cardio embolism as the major common subtype.

Hypertension is the most prevalent vascular risk factor (83,4%), followed by dyslipidemia (50,6%) and diabetes mellitus (48,5%), similar to other studies by Marrone et al. [22] and Bahou et al. [25]. In this study, hypertension was only statistically significant in SVD and undetermined etiology subtypes. This result is similar to a study conducted by Arboix et al. which stated that hypertension is independently associated with SVD, along with diabetes [28]. From multivariate analysis, it was shown that patients with hypertension have three times higher risk of SVD (OR 3; CI 1,12-8,05). Hypertension causes SVD by changing brain vasculature through fibrinoid deposition in the small vessel and hypertrophy of smooth muscle, thus leading to brain ischemia in affected areas [29]. Other condition thought to be associated with SVD and undetermined etiology subtype is nocturnal hypertension which is affected by circadian rhythm. Nocturnal blood pressure variability may be associated with wake-up stroke incidence which occurs in 20% to 30% of ischemic stroke [30].

Diabetes mellitus is also recognized as a risk factor for LAA and SVD. Several studies reported that diabetes is associated with SVD more strongly than other subtypes [26, 31], including a study by Grau et al. in which diabetes prevalence was 35,5% in SVD compared to 29% in LAA and 28% in cardioembolism [15]. However, in our study, diabetes mellitus was significantly more prevalent in LAA (n=77, p=0,016). It is well known that chronic hyperglycemia will lead to macrovascular complication due to endothelial dysfunction, inflammation, and acceleration of atherosclerotic process as the effect of increased oxidant production, lipoprotein enrichment, and Advanced Glycosylation End Products (AGEs) formation [32–34]. However, previous study suggested that pathogenesis of macrovascular and microvascular complication occurs simultaneously which explain why ischemic stroke in diabetes can occur due to macro- or microvascular complication [34]. On the other hand, although the prevalence of dyslipidemia was relatively high (50,6%), it had no statistical significance as a risk factor among five ischemic stroke subtypes. This was similar with result of Zafar et al. [26] and Turin et al. studies [18].

Ischemic heart disease (75%) and atrial fibrillation (50%) were the major risk factors for cardioembolic subtype, according to Nadia et al. [35]. In our cardioembolic subtype patients, atrial fibrillation was more frequent than myocardial infarction with prevalence 60% and 20%, respectively. Atrial fibrillation had significant association in cardioembolic subtype with p<0,001. However, the wide CI 95% of atrial fibrillation in cardioembolic subtypes (CI 95% 13,6-946,5) may be caused by low number of cases. Low number of cardioembolic cases in our study may happen due to high mortality rate of cardioembolic stroke [36]; therefore many patients might have been died in secondary hospital or out of our hospital. Atrial fibrillation causes contractile dysfunction and static blood flow leading to thrombus formation and brain embolism. In addition, the risk of thromboembolism increases as atrial cardiopathy worsens due to structural remodeling of the atrium [37].

A major limitation of our retrospective study was the certainty of appropriate diagnostic evaluation because it was only obtained from medical records. In addition, our results only reflect the relationship of risk factors on each subtype since we had no control group (without stroke). Risk factors analyzed in this study were limited to hypertension, diabetes mellitus, dyslipidemia, atrial fibrillation, and myocardial infarction even though there are other risk factors that should be accounted for such as smoking history, family history of stroke, and BMI. Therefore, further large and multicenter prospective study is needed to better understand the ischemic stroke subtypes proportion and its risk factors in Indonesia.

## 5. Conclusion

The most common etiology of ischemic stroke in our study was large-artery atherosclerosis. There were differences of risk factors between the stroke subtypes. We found that diabetes was significantly associated with large artery atherosclerosis while hypertension was associated with small vessel disease and undetermined etiology subtypes. Moreover, atrial fibrillation was associated with cardioembolic subtypes. Further studies are required to understand epidemiology of ischemic stroke subtypes and its relationship with the risk factors.

## Figures and Tables

**Table 1:** Characteristics of the subjects (n=235).

**Characteristic**	**n (**%**)**	**Mean (SD)**
**Demographics**		
Male	139 (59,1)	
Female	96 (40,9)	
Age >65 years	82 (34,9)	60,9 (11,4)
**Risk factors**		
Hypertension	196 (83,4)	
Diabetes mellitus	114 (48,5)	
Dyslipidemia	119 (50,6)	
Myocardial infarction history	13 (5,5)	
Atrial fibrillation	6 (2,6)	
**TOAST classification**		
Large artery atherosclerosis	140 (59,6)	
Small vessel disease	65 (27,7)	
Cardio embolic	5 (2,1)	
Other determined etiology	2 (0,9)	
Undetermined etiology	23 (9,8)	

**Table 2:** Risk factors among ischemic stroke subtypes.

Risk factors	Large artery atherosclerosis		Small vessel disease		Cardio embolic		Other etiologies		Undetermined etiology	
	n (%)	*p*-value	n (%)	*p*-value	n (%)	*p*-value	n (%)	*p*-value	n (%)	*p*-value
Males	85 (60,7)	0.55^†^	40 (61,5)	0,64^†^	3 (60)	0,67^‡^	0 (0)	0,17^‡^	11 (47,8)	0,25^†^
Females	55 (39,7)		25 (38,5)		2 (40)		2 (100)		12 (52,2)	
Age > 65 years	53 (37,9)	0,25^†^	20 (30,8)	0,41^†^	2 (40)	0,57^‡^	0 (0)	0,42^‡^	7 (30,4)	0,64^†^
Hypertension	118 (84,3)	0,66^†^	60 (92,3)	**0,023** ^†^	4 (80)	0,6^‡^	1 (50)	0,3^‡^	13 (56,5)	**<0,001** ^†^
Diabetes	77 (55)	**0,016** ^†^	27 (41,5)	0,19^†^	0 (0)	0,06^‡^	1 (50)	0,74^‡^	9 (39,1)	0,34^†^
Dyslipidemia	72 (51,4)	0,77^†^	32 (49,2)	0,79^†^	0 (0)	0,028^‡^	1 (50)	0,74^‡^	14 (60,9)	0,3^†^
MI	9 (6,4)	0,46^†^	2 (3,1)	0,31^†^	1 (20)	0,25^‡^	0 (0)	0,89^‡^	1 (4,3)	0,79^†^
AF	3 (2,1)	0,46^‡^	0 (0)	0,14^‡^	3 (60)	**<0,001** ^‡^	0 (0)	0,95^‡^	0 (0)	0,41^†^

^†^Chi-square test; ^‡^Fisher's exact test.

**Table 3:** Multivariate analysis in small vessel disease.

Risk factors	Small Vessel Disease		Risk factors	Cardioembolism	
	P value	OR (95% CI)		P value	OR (95% CI)
Hypertension	0.029	3 (1.12 – 8.05)	Atrial Fibrillation	<0,001	113,5(13,6-946,5)
Diabetes	0.220	0.69 (0.39 – 1.24)	Myocardial Infarction	0,478	3,02 (0,14-64,1)

## Data Availability

The journal articles used to support the findings of this study are included within references in the article and freely available in each journal website.
